# Compared EC-AFM Analysis of Laser-Induced Graphene and Graphite Electrodes in Sulfuric Acid Electrolyte

**DOI:** 10.3390/molecules26237333

**Published:** 2021-12-02

**Authors:** Claudia Filoni, Bahram Shirzadi, Marco Menegazzo, Eugenio Martinelli, Corrado Di Natale, Andrea Li Bassi, Luca Magagnin, Lamberto Duò, Gianlorenzo Bussetti

**Affiliations:** 1 Department of Physics, Politecnico di Milano, p.za Leonardo da Vinci 32, I-20133 Milan, Italy; bahram.shirzadi@mail.polimi.it (B.S.); marco.menegazzo@mail.polimi.it (M.M.); lamberto.duo@polimi.it (L.D.); gianlorenzo.bussetti@polimi.it (G.B.); 2 Department of Electronic Engineering, University of Rome Tor Vergata, v. del Politecnico, I-00133 Rome, Italy; martinelli@ing.uniroma2.it (E.M.); dinatale@eln.uniroma2.it (C.D.N.); 3 Department of Energy, Politecnico di Milano, v. Ponzio 34/3, I-20133 Milan, Italy; andrea.libassi@polimi.it; 4 Department of Chemistry, Materials and Chemical Engineering “Giulio Natta”, Politecnico di Milano, v. Mancinelli 7, I-20131 Milan, Italy; luca.magagnin@polimi.it

**Keywords:** laser-induced graphene, Kapton, graphite foils, graphite, EC-AFM, Raman spectroscopy

## Abstract

Flexible and economic sensor devices are the focus of increasing interest for their potential and wide applications in medicine, food analysis, pollution, water quality, etc. In these areas, the possibility of using stable, reproducible, and pocket devices can simplify the acquisition of data. Among recent prototypes, sensors based on laser-induced graphene (LIGE) on Kapton represent a feasible choice. In particular, LIGE devices are also exploited as electrodes for sensing in liquids. Despite a characterization with electrochemical (EC) methods in the literature, a closer comparison with traditional graphite electrodes is still missing. In this study, we combine atomic force microscopy with an EC cell (EC-AFM) to study, in situ, electrode oxidation reactions when LIGE or other graphite samples are used as anodes inside an acid electrolyte. This investigation shows the quality and performance of the LIGE electrode with respect to other samples. Finally, an ex situ Raman spectroscopy analysis allows a detailed chemical analysis of the employed electrodes.

## 1. Introduction

The development of portable, low-cost, and effective devices has been increasing over the past few decades [1,2]. Among the different applications, electrochemical biosensors, e.g., neurotransmitters or non-enzymatic glucose sensors, have received large interest due to their high sensitivity and selectivity, easy miniaturization, efficiency, and ease of use [3,4,5,6,7,8,9,10,11]. The commercial versions of combined electrode systems are typically based on carbon or carbon-based materials (e.g., glassy carbon, carbon nanosheet electrodes, etc.) that have good electrical properties and are very handy materials [12,13,14]. These systems are usually modified with metal nanoparticles, carbon nanotubes, or graphene in order to reach the best parameters, such as faster electron transfer kinetics, low residual current, and excellent thermal conductivity [15,16]. Supercapacitors have a long history that has seen the employment of various carbon-electrode-based solid-state devices up to the development of their miniaturized form, known as micro-supercapacitors [17]. The rapid growth of portable electronics, remote control systems, radio-frequency detectors, and micro-electro-mechanical systems has significantly increased the need for device miniaturization [18,19,20]. Currently, their potential is becoming more evident with respect to a fast charging–discharging rate, a long cycling life, flexibility, and shape diversity, together with their functional convenient size and their lightweight [21,22]. In addition, global climate change calls for the urgent development of carbon-neutral and renewable-energy rechargeable devices, such as batteries, and hybrid technologies, including electric vehicles [23,24,25,26].

One of the current trends foresees the exposure of carbonaceous substrates to intense laser beams, which both reduces substrate thickness (down to a multi-layered graphene system) and increases the porous density of the original structure (three-dimensional (3D) porous graphene). In fact, the intense laser beam burns the pristine substrate and removes part of its mass. At the same time, the surface graphitization increases the number of defects, holes, and pores or, generally speaking, the surface’s roughness [27,28,29]. This process acts as one-step, scalable printing to produce carbon-based electrodes patterning on a stretchable and flexible platform [30]. It is widely known how the electric properties of graphene change depending on the stacking of layers [31,32]. Graphene is a one-atom-thick, bi-dimensional (2D) carbon sheet with carbon atoms arranged in a honeycomb lattice. The hexagonally *sp*^2^-bonded carbon atoms arrange to reach very high thermal conductivity (≈5000 Wm^−1^K^−1^), mobility (≈10,000 cm^2^V^−1^s^−1^), and surface area (2630 m^2^g^−1^). With the aim of forming sensing prototypes, some of the other primary advantages of graphene include a high Young’s modulus and high optical transparency in the visible wavelength range [33,34]. Three-dimensional, or multilayered, graphene is a sort of compromise between thick graphite and a single graphene layer that, however, can be exploited for producing portable and flexible energy storage devices. In particular, 3D porous graphene presents better mechanical stability and higher catalytic activity [35]. It is well known from the literature that graphene can be fabricated by a large variety of methods, such as chemical exfoliation of graphite and chemical vapor deposition (CVD). Nevertheless, these production techniques can be quite expensive or can have some disadvantages: CVD needs a high amount of energy, and the mechanical exfoliation process yields a low-quality graphene product with some forms of defects, e.g., microscopic corrugation [36]. Moreover, the carbon-based electrochemical devices available in the market are normally fabricated by lithography, e.g., photolithography, dry etching, or film transfer; all these techniques have a high production cost and require consistent time-consuming procedures [37,38]. To make the best laser-pattern process, it is usually necessary to combine a conductive matrix, e.g., graphene, with suitable flexible material as the substrate, such as polyurethane or polyimide, to form a novel composite [39].

In this regard, in 2014, laser-induced porous 3D graphene was synthesized on polymers by Lin et al. [40]. In this novel one-step process, the porous graphene film was patterned by pointing a CO_2_ laser onto a commercial polyimide foil in an ambient environment. No baking step nor need of materials, other than polyimide foil, were required [41,42]. Among the different carbon-containing materials, the first material used as a precursor was Kapton, a classic polyimide developed in the 1960s by DuPont. Polyimides (C_22_H_10_O_5_N_2_) have been massively produced since 1955; they are lightweight, flexible, resistant to heat and chemicals, and they show natural structural anisotropy due to the preferential orientation of the polymer chains obtained during the manufacturing process. Kapton exhibits excellent physical properties and is exploited as an electrical and thermal insulator in harsh environments [43]. In the photothermal conversion process, lasing causes the propagation of a high degree of lattice vibrations through the precursor material, resulting in localized heating; meanwhile, *sp*^3^ carbon atoms are converted into a *sp*^2^ hybridization and C−N, C−O, C−H, and C=O bonds dissociate [44]. Laser-induced graphene’s (LIGE) foam-like morphology has a direct influence on electron transfer kinetics enhancement (increased surface-to-volume ratio): The 3D porosity formed during the laser scribing process contributes to faster electron transfer rates [45,46]. The resulting product (LIGE) shows similar properties as commercial graphene but a higher porosity with respect to pristine graphene, which eases the adsorption of ions, influencing the sensitivity and selectivity of the systems [47].

The aim of this study is to compare the behavior of traditional electrodes, such as graphite electrodes (namely, Highly Oriented Pyrolytic Graphite (HOPG), graphite foil, and glassy carbon) and the innovative LIGE electrode. The electrode behavior was studied when specimens were immersed in a 1 M sulfuric (H_2_SO_4_) electrolyte in which the main electrochemical characteristics in graphite electrodes are known. In order to achieve this goal, by combining an electrochemical Atomic Force Microscope (EC-AFM) and ex situ Raman spectroscopy measures, we performed a detailed analysis of the employed electrodes in a liquid environment to study the electrode oxidation reaction and compared the obtained results.

## 2. Results and Discussion

### 2.1. EC Characterization

**Figure 1:**
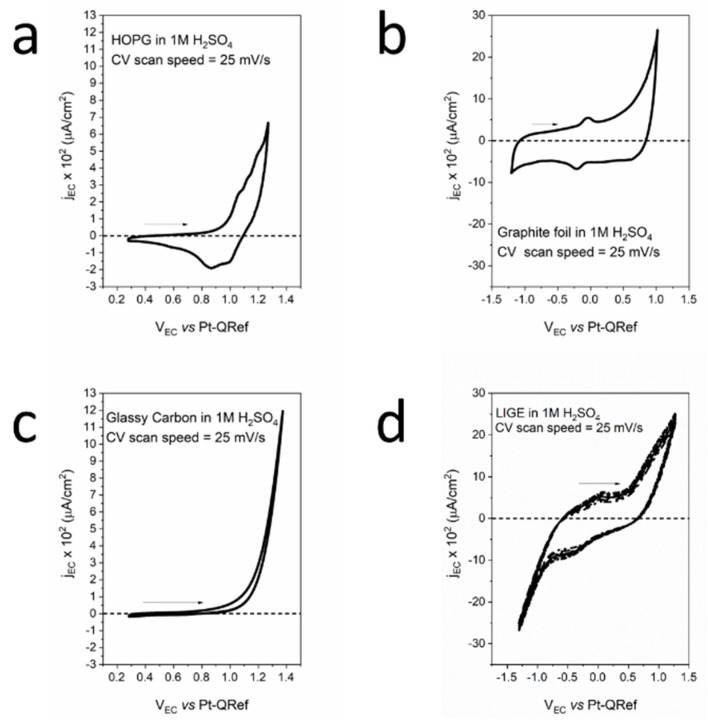
Cyclic-voltammetry acquired on the four exploited electrodes: (**a**) HOPG; (**b**) graphite foil; (**c**) glassy carbon; and (**d**) LIGE.

In Figure 1, we report the EC analysis of our electrodes when immersed in 1 M H_2_SO_4_ electrolytes. The panel shows the acquired cyclic-voltammetry (CV), i.e., a voltammogram of the flowing faradaic current (*y*-axis) when a potential (*x*-axis) is applied to the sample (working electrode, WE).

Figure 1a refers to the well-studied HOPG CV, where shoulders appear as soon as a higher EC potential with respect to the oxygen evolution reaction (OER) is applied to the WE [48]. These features are related to the solvated anion intercalation inside the graphite basal plane. The anodic exchange charge is about 20 μC. During the cathodic potential sweep, a negative current is measured. The latter is traditionally referred to as a partial de-intercalation process that is probably coupled with other processes still under discussion [49]. Anion intercalation changes graphite crystal structures because solvated anions are tidily interposed between the graphite layers (graphite intercalated compound, GIC) [50]. The overall interpretative model was developed by Goss and co-workers at the beginning of the 1990s [51]. According to this model, solvate anions can diffuse inside HOPG-stratified crystal structures through surface defects, such as steps and holes. However, following recent results obtained in our research, acid electrolytes are able to dissolve the graphite basal plane, thus increasing the defects and allowing solvated anions to easily enter the HOPG bulk [48]. When the OER is reached, gases (namely, CO, CO_2_, and O_2_) also evolve inside the HOPG’s bulk. Here, they can produce intense pressures that are able to swell the basal plane and create blisters on the surface. As a consequence of the detailed knowledge of these processes in HOPG electrodes, we propose this electrode dynamic as a model to interpret results collected on other carbonaceous samples [52].

When graphite foil (GF) was employed, the faradaic current intensity was higher, which is reasonably due to the presence of many defects on the GF surface (see Figure 1b). These defects are preferential sites for electrolyte intercalation. In addition, the effective electrode roughness was higher with respect to the HOPG’s basal plane and, consequently, the electrode surface was exposed to the electrolyte. We observed that two peaks (oxidation and reduction) were placed between −0.25 V and 0.0 V (vs. PtQRef). The exchanged anodic (cathodic) charge was approximately 180 μC (240 μC), almost an order of magnitude higher with respect to the anodic charge exchanged in the HOPG intercalation process. These voltammetric features, which can be attributed to the presence of oxygen compounds, intruded the GF sample through possible pores [53]. The intense faradaic current during the anodic evolution suggests possible anion intercalation inside the foil. However, the cathodic sweep was significantly different with respect to the HOPG voltammogram. In contrast to the HOPG electrode, gases produced at the OER can be eliminated through the pores and defects present in the GF.

Glassy carbon (GC) was produced from pyrolysis of furfuryl alcohol, phenolic resins, and similar polymers under heating up to 3000 °C. The obtained system was not graphitizable, i.e., it was not possible to convert the GC into crystalline graphite. The sample combined mechanical properties close to those of ceramics and glass. The local structure was made up almost completely of *sp*^2^-bonded carbon atoms. As a consequence of the GC vitreous behavior, the EC performance of this electrode demonstrated faradaic current intensity enhancement due to the OER without any other feature or the presence of a negative (cathodic) current (see Figure 1c). In these conditions, anion intercalation was precluded, and the voltammogram showed an almost superimposable anodic and cathodic sweep. No de-intercalation features were detected at negative faradaic currents, confirming the picture of a very inert electrode.

The LIGE electrode CV is shown in Figure 1d. Interestingly, we observed a partial similarity with the GF CV. In fact, two features, placed between −0.5 V and 0.0 V, appeared in the CV. The anodic (cathodic)-exchanged charge was approximately 390 μC (264 μC), about a factor of 2 higher with respect to the GF. Considering both these similarities and the high surface roughness (a consequence of the Kapton burning process), we speculate that the GF CV interpretation, in terms of a significant presence of oxygen compounds, is also reasonable for the LIGE electrode. The similarity of the LIGE voltammogram with that reported for the GF suggests that, as in the previous case, anion intercalation enhances the faradaic current during the anodic sweep, but the high porosity and defect density allow gases to move out from the buried LIGE layers, without any particular signature in the voltammogram. Finally, we observed that, in Figure 1d, four CVs were reported. Their perfect superposition directly shows the stability of the LIGE electrode during subsequent EC treatments.

### 2.2. EC-AFM Characterization

**Figure 2:**
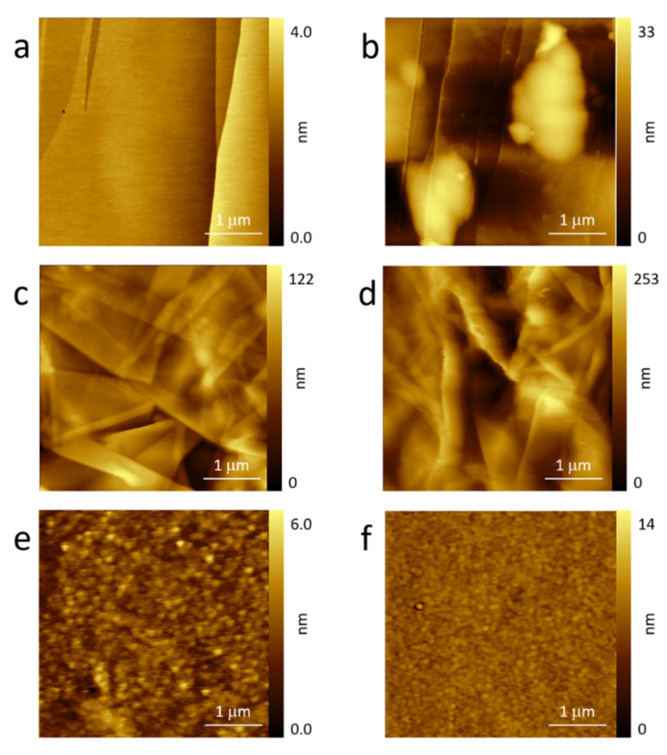
AFM images of the used electrodes before (HOPG (**a**); GF (**c**); GC (**e**)) and after (HOPG (**b**); GF (**d**); GC (**f**)) the EC treatment.

In Figure 2, we report the topographic images acquired for HOPG (panels Figure 2a,b), GF (Figure 2c,d), and GC (Figure 2e,f) electrodes. Panels a, c, and e refer to pristine electrodes while the other panels refer to the sample after one CV in sulfuric acid electrolyte (see Figure 1).

HOPG (see Figure 2a) was characterized by flat terraces and sharp step edges. The situation significantly changed after the intercalation process [50]: Clear blisters (Figure 2b), a consequence of the entrapped gas deformation of the basal plane, were visible close to or on the step edges. The latter were preferential regions of solvated ion intercalation [50,51].

GF is shown in Figure 2c. The surface morphology appeared as the superposition of different thin graphite layers. Many steps, defects, and pores were observed in the image. Consequently, the expected gas evolution during the anodic sweep in acid electrolytes could find more paths for its outflow, as discussed above. This hypothesis is well supported by the AFM image acquired after the CV treatment (see Figure 2d). The roughness undoubtedly increased, probably due to swelling of some GF regions, but blisters (at least, as defined for HOPG) were not clearly visible in the acquired topography. The general behavior recalls a carbonaceous surface after a partial dissolution. This process is indeed observed on HOPG and reported by the authors [48].

The GC electrode was very stable. The surface topography did not show significant changes between the pristine and used electrode (see Figure 2e,f). The former was characterized by relatively small particles and valleys that were randomly distributed as a consequence of the GC industrial production. The electrode’s surface, after the EC treatment, appeared more uniform in which particles did not have a clear contrast. This effect was obviously due to the sulfuric acid electrolytes but, when working as an electrode, the GC surface did not show blisters, swelled areas, or holes as a consequence of the carbon dissolution, as already reported by the authors [48,52]. This result is reasonable when considering the mechanical properties of the GC as reported above.

**Figure 3:**
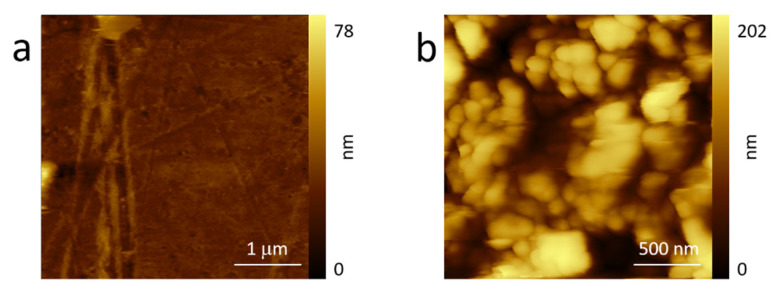
AFM images of the pristine Kapton (**a**) and after laser burning (**b**) acquired in air. See the text for more details.

LIGE required a different morphological analysis. In fact, while Kapton can be characterized by AFM, the LIGE electrode presented many difficulties due to the high surface roughness. For this reason and in view of completeness, we decided to compare Kapton morphology (see Figure 3a) with its topography after laser burning (b). In order to avoid very high rough regions, the reported AFM image (Figure 3b) refers to an area where the original Kapton was still partially visible.

Kapton morphology (Figure 3a) was basically flat, even if it were possible to observe scratches or bumps that are possible after industrial production. The topography of the partially burnt sample significantly changed: the roughness immediately increased, and AFM images were possible only at reduced areas (2 × 2 μm^2^). Here, many roundish objects characterized the surface. The morphology was compatible with processes of polymer laser melting, which can sometimes produce periodic structures [54]. From this result, it is clear that an AFM acquisition of the total burnt Kapton sample was not possible. However, this analysis suggests that the expected increased number of defects and pores precludes any gas encapsulation (blister evolution) during the anodic sweep, which is in close agreement with the GF behavior.

### 2.3. Raman Spectroscopy Characterization

**Figure 4:**
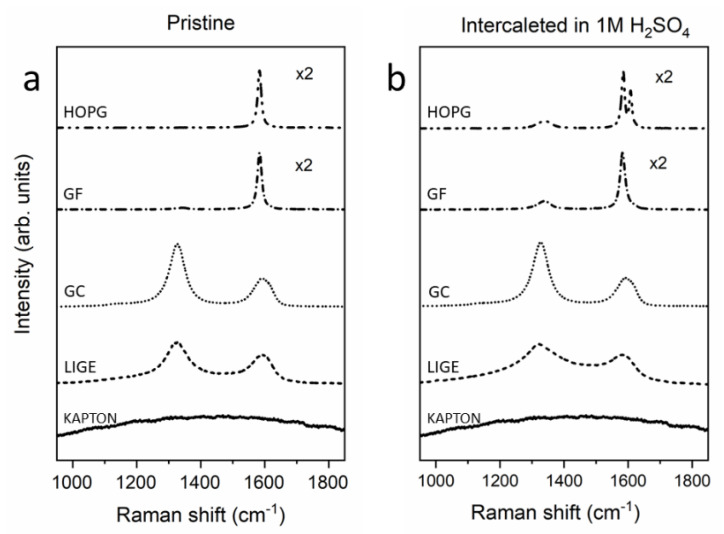
Raman spectra of electrodes in pristine condition (**a**) and after the EC treatment (**b**).

In Figure 4, we show the Raman spectra of the electrodes before (Figure 4a) and after (Figure 4b) the electrochemical process in 1M H_2_SO_4_. According to the literature [49,55], the pristine HOPG spectrum, the main reference for Raman spectroscopy evolution as a function of the EC treatment, highlights only one peak (G-peak) at approximately 1581 cm^−1^ (Figure 4a). The presence of only the G-peak is a consequence of a carbon crystal possessing *sp*^2^ C–C bonds.

Surprisingly, the pristine GF Raman spectrum is mainly characterized by only the G-peak. Only a smaller feature, placed at approximately 1330 cm^−1^, appears in the data. The latter, when investigated in other carbon compounds (see below), is labeled as the D-peak, and it is generally related to the presence of structural alterations in the graphitic structure. As a consequence, despite having more steps and defects that can enhance the faradaic current in CV (see above), the GF is characterized by a local structural behavior superimposable to the HOPG structure. Considering that the GF production foresees the mechanical compression of HOPG grains, the GF Raman spectrum reported in Figure 4a is expected.

Conversely, the GC sample is prepared by following a high-temperature process (see above) that significantly enhances the crystal structure. The Raman spectrum is, thus, characterized by a significant D-peak for which its intensity is higher than the G-peak. In addition, the latter feature is also broader with respect to the crystalline HOPG spectrum, in agreement with the GC structural properties.

The Kapton Raman spectrum did not show any signal in the region of interest while, when burned by the laser beam (LIGE), two features appeared in the same spectrum positions of the G-peaks and D-peaks. The close agreement between the GC and LIGE Raman spectra suggests that the burned Kapton is characterized by a disordered graphitic structure for which its D-peak has a higher intensity with respect to the G-peak and the latter has a wider line shape with respect to the crystalline HOPG sample.

The HOPG electrode was analyzed by Raman spectroscopy after EC treatment (see Figure 4b). Following the results reported by the authors in reference [49], the evolution of blisters and the general detriment of graphite basal plane allow the evolution of a D-peak (smaller with respect to the G-peak) and a second feature (G_i_) at higher Raman shift (about 1610 cm^−1^) strictly related to the chemical action of the intercalated acid. The G_i_ peak is a specific characteristic of the intercalated HOPG electrode and was not observed in any other sample.

The effect of the EC treatment was also clearly visible on the GF sample. While the G-peak seemed to be almost unaltered, the D-peak was more pronounced in the spectrum, suggesting that part of the GF surface underwent a detriment that was also visible in the AFM image (see Figure 2d).

The non-reactive behavior of the GC electrode was proved by the Raman analysis. A fast comparison between Figure 4a,b revealed that the spectra were superimposable. The stability of the GC sample was also confirmed by previous topographic analyses and justifies the acquired CV in Figure 1.

The LIGE electrode showed modified features after the EC treatment. In contrast to the GC electrode, the D-peak of the LIGE sample became broader with respect to the pristine sample, suggesting some changes (chemical and/or physical, as a disorder enhancement) in the burned Kapton region. The signal-to-noise ratio decreased substantially probably due to the fact that the uppermost part of the LIGE was mechanically removed after immersion inside the EC cell.

## 3. Materials and Methods

Different samples [(25 × 25 × 1) mm^3^] have been exploited in this work. Their active surface, when used as electrodes, is 0.2 cm^2^ in order to avoid high faradaic current intensities:

(i) The Z-grade highly oriented pyrolytic graphite (HOPG) was acquired from Optigraph and was mechanically exfoliated before each experiment by means of an adhesive tape.
(ii) Graphite foil and glassy carbon samples were provided by Goodfellow. The samples were cleaned in ethanol and deionized water before their employment.
(iii) A Trotec Speedy 100 laser cutter (Trotec Laser Inc., Marchtrenk, Austria) was used for producing porous graphene films using Kapton, a commercial polymer film [40]. Graphene production was a result of the conversion of the *sp*^3^ carbon atoms in the Kapton into *sp*^2^ carbon atoms by pulsed laser irradiation. Laser cutter parameters are found in reference [56].

The electrolyte was prepared by diluting a H_2_SO_4_ solution (95–97% *w*/*w*, Merck Darmstadt, Germany) to obtain a 1 M concentration. Before filling the EC cell, the electrolyte was de-aerated by bubbling pure Ar in a Squibb separator funnel for some hours.

The homemade EC cell was made in Teflon and was placed above the sample, which was the working electrode (WE) of our system. In order to avoid solvent leakages, a Viton O-ring was interposed between the cell and the sample.

In addition to the WE, two Pt wires were inserted inside the EC cell: one wire, which turns around the overall cell, played the role of the counter electrode (CE) while the head of the second wire was used as a reference electrode (RE). More precisely, the latter was a quasi-reference because it did not exploit a redox couple. Nonetheless, the platinum quasi-reference (Pt-QRef) ensures good stability (within a few mV) when immersed in acid electrolytes, and a stable EC potential shift (+740 mV) with respect to the standard hydrogen electrode [50].

AFM images were collected by a commercial Keysight 5500 model. According to the sample’s surface roughness and/or solid electrolyte interface evolution, the selected acquisition mode was contact or non-contact. In the former case, a constant laser deflection was used during the tip approach while, for the latter, a resonance frequency of about 130 kHz was found.

Raman spectroscopy was conducted ex situ before and after EC analysis. The samples were placed under a Nikon Eclipse Ni microscope of an NT-MDT Confotec NR500 confocal Raman spectrometer. A 632.8 nm laser (of approximately 23 mW) was exploited as an excitation source. The light was focused by a 100× objective directly on the sample. Here, the measured laser power was 5 mW because of multiple reflections of the laser beam on mirrors. Moreover, we carefully checked that no heating or surface damages to the samples were induced by the laser. The spectra were recorded with multiple scans, integrating the signal for an overall time that changed for each sample in order to optimize the spectrum.

## 4. Conclusions

Laser-induced graphene film on Kapton substrate represents an innovative, economic, and technologically important procedure for obtaining conductive circuits or electrodes on flexible materials (such as Kapton foils). In the Introduction of this study, we summarized the wide literature produced in recent years where these innovative devices have been successfully employed. Kapton foils have also found applications as electrodes (LIGE), and their EC characterization has been reported in the literature. Despite this fact, a comparative analysis of the LIGE performances with respect to other carbon-based electrodes is still missing. In this study, we reported an EC, microscopic (namely AFM), and spectroscopic (Raman technique) investigation of four electrodes (HOPG, graphite foil, glassy carbon, and LIGE) when immersed and used in a sulfuric electrolyte. HOPG is considered a model system because the intercalation processes observed on the electrode surface, when the oxygen evolution reaction is reached, are well reported in the literature and by the authors. Glassy carbon is a widely used electrode by comparison, due to its mechanical and chemical stability in corrosive environments. On the other hand, graphite foil has been exploited as a technological electrode in modern Li-batteries since its production is quite economic but, more importantly, it is a flexible electrode. LIGE was then compared to the above-mentioned electrodes to test its characteristics in analogous conditions.

The result of our study, summarized in Table 1, proves the stability of LIGE in acid electrolytes, a similarity in CV analysis (in particular with the graphite foil), and Raman features that recall what was observed on the glassy carbon electrode. In summary, LIGE collects the important and significant behaviors of the other carbonaceous electrodes and thus represents a concrete alternative in many innovative applications.

## Figures and Tables

**Table 1:** Comparison table between HOPG, GF, GC and LIGE features.

Electrode	Cyclic-Voltammetry	AFM	Raman
HOPG	intercalation and de-intercalation features	blister evolution after anion intercalation	evolution of D and G_i_ peaks after the EC treatment
GF	oxidation and reduction peaks	surface swelling and pores	only D peak appearance after the CV
GC	no specific features	no significant changes related to the EC treatment	stable D and G peaks before and after the EC treatment
LIGE	oxidation and reduction peaks	too rough surface	stable D and G peaks before and after the EC treatment
